# Further validation of the Cognitive Biases Questionnaire for psychosis

**DOI:** 10.1186/s12888-022-04203-8

**Published:** 2022-08-19

**Authors:** Crystal Samson, Amélie M. Achim, Veronik Sicard, Andy Gilker, Audrey Francoeur, Nicolas Franck, Briana Cloutier, Charles-Edouard Giguère, Francelyne Jean-Baptiste, Tania Lecomte

**Affiliations:** 1 Département de Psychologie, Laboratoire d’étude sur la schizophrénie et les psychoses orienté vers l’intervention et le rétablissement Pavillon Marie-Victorin, Université de Montréal, 90 Vincent D’Indy Ave, Outremont, QC, H2V 2S9 Canada; 2 Centre de recherche de l’Institut Universitaire en Santé Mentale de Montréal (CR-IUSMM), Québec, Canada; 3 Université Laval, Québec, Canada; 4 Centre de recherche CERVO, Québec, Canada; 5 Centre de recherche en santé durable VITAM, Québec, Canada; 6 Children’s Hospital of Eastern Ontario Research Institute, Ottawa, Canada; 7 Département de Génie biotechnologique, Université de Sherbrooke, Québec, Canada; 8 Faculté de Médecine Lyon-Sud Charles Mérieux, Université Claude Bernard Lyon 1, Lyon, France; 9 Pôle Centre rive gauche & Centre ressource de réhabilitation psychosociale, Centre hospitalier Le Vinatier, Lyon, France; 10 Centre National de la Recherche Scientifique (CNRS), Bron, France

**Keywords:** Cognitive biases, Psychosis, Depression, Schizophrenia, Validation, Questionnaire

## Abstract

**Background:**

Cognitive biases are recognized as important treatment targets for reducing symptoms associated with severe mental disorders. Although cognitive biases have been linked to symptoms in most studies, few studies have looked at such biases transdiagnostically. The Cognitive Bias Questionnaire for psychosis (CBQp) is a self-reported questionnaire that assesses cognitive biases amongst individuals with a psychotic disorder, as well as individuals with other severe mental disorders. The current study aims to validate a French version of the CBQp and to explore transdiagnostic cognitive biases in individuals with psychotic disorders, individuals with depression, and in healthy controls.

**Methods:**

The CBQp was translated into French following a protocol based on international standards. Discriminant validity and internal consistency were determined for total score and each subscale score. Confirmatory factor analyses were performed to test construct validity. Finally, cluster analyses were conducted to investigate cognitive biases across diagnostic groups.

**Results:**

Our results were similar to those of the original authors, with the one-factor solution (assessment of a general thinking bias) being the strongest, but the two-factor solution (assessing biases within two themes relating to psychosis) and the five-factor solution (assessment of multiple distinct biases) being clinically more interesting. A six-cluster solution emerged, suggesting that individuals with similar diagnoses score differently on all cognitive biases, and that individuals with different diagnoses might have similar cognitive biases.

**Conclusions:**

The current findings support the validity of the French translation of the CBQp. Our cluster analyses overall support the transdiagnostic presence of cognitive biases.

**Supplementary Information:**

The online version contains supplementary material available at 10.1186/s12888-022-04203-8.

## Background

A transdiagnostic approach to mental health research has been recommended by The National Institute of Mental Health (NIMH) [1] and is becoming increasingly popular amongst researchers. The current categorical diagnostic system is designed to facilitate the communication of information regarding epidemiology, clinical descriptions, pathogenesis, treatment options, and prognosis and outcome among treatment providers, patients, families, and the public [2]. Regarding schizophrenia and depression, Mellsop & al [2]. consider that the current classification fails to meet its objectives. Categorical approaches are widely criticized by both researchers and health professionals [2–4] for a plethora of reasons.

First, diagnostic criteria currently used to differentiate psychiatric disorders are solely based on symptomatology; however, identical symptoms are included as core criteria for different psychiatric disorders diagnoses. Indeed, one can observe a depressed mood in individuals with major depressive disorder, but also in those diagnosed with psychotic depression, bipolar disorder, schizoaffective disorder, or schizophrenia. Importantly, many of the negative symptoms of schizophrenia (e.g., social withdrawal, anhedonia) are considered symptoms of depression in mood disorders. Second, some diagnoses seem to fit even less in a single category. For instance, criteria for schizoaffective disorder diagnosis are among the most criticized criteria as they encompass the same symptoms as bipolar disorder, and only differ in the duration of psychotic symptoms in relation to the mood symptoms. Third, given the plurality of possible symptom profiles for a given diagnosis, it is possible for two individuals to share a diagnosis, yet exhibit highly different symptoms [5, 6]. Fourth, severe psychiatric disorders do not have a single etiology. For example, a recent genome-wide association study revealed more than 200 common risk variants for schizophrenia [7]. Moreover, studies suggest that severe psychiatric disorders may be genetically linked [8] and that many genes linked to risk for psychiatric disorders may not be diagnostically specific in their effect. Indeed, meta-analyses showed that variants on a single gene (the 5-HT2A receptor) are linked to three different disorders (schizophrenia, bulimia, and anorexia nervosa). For instance, overlapping genes on chromosome 13q (termed G30 and G72) may be associated with both schizophrenia and bipolar disorder [9], and several popular candidate genes (e.g., serotonin transporter, dopamine transporter, dopamine 2 receptor) are significantly associated with a wide variety of psychiatric disorders or psychiatrically relevant traits [10, 11]. Studies also suggest that different psychiatric disorders may be precipitated by similar environmental factors [12, 13]. Exposure to childhood adversity is a good example, as it is linked to higher rates of multiple observed disorders [14]. Risks factors for severe psychiatric disorders are thus not related to the manifestation of a particular disorder, but rather to the likelihood of developing a severe mental disorder in general. Moreover, traditional classification systems that frame mental disorder diagnoses as independent entities fail to consider high rates of observed comorbidity [15] like depression and anxiety [16], or schizophrenia and social anxiety [17]. Finally, responses to various treatments appear to be specific to the symptoms that are targeted and not to the diagnosis itself, with symptoms intensity being a better predictor of treatment needs than the given diagnosis [18]. Empirically-based psychotherapeutic interventions aim to alter the dysfunctional thought patterns and cognitive biases underlying specific symptoms and are not diagnosis-specific [13].

Mathews and MacLeod [19] defined cognitive biases as the tendency to process information in ways that favor certain types of emotional meaning or valence. We can distinguish three categories of cognitive biases: attentional biases, interpretation biases, and memory biases [19, 20]. Interpretation biases are the tendencies to interpret or infer ambiguous information according to a certain emotional meaning or valence [19].

Cognitive biases, especially interpretation biases, are recognized as important treatment targets for reducing symptoms associated with severe mental disorders and Cognitive-oriented psychotherapies are largely based on the assumption that cognitive biases are causally related to symptoms [19]. Indeed, studies show that it is possible to reduce symptoms, including depressive and psychotic ones, as well as prevent relapses by targeting cognitive biases [19].

Cognitive behavioral therapy (CBT) is an evidence-based therapy [21, 22] that focuses on the relationship between cognitions, emotions, and behavior. A recent metaanalysis of CBT randomized controlled trials (RCT) showed that heterogeneity between RCT was low and that CBT remained effective across different conditions [23].

CBT for psychosis (CBTp) is also effective [24–29]) in reducing psychotic symptoms relapse at 12 months and improving functioning. CBTp is recommended by several clinical guidelines [30, 31] for severe mental illness to diminish distress or symptoms linked to psychotic disorders.CBTp and aims to modify, amongst other things, beliefs underlying an individual’s hallucinations and delusions by targeting the cognitive biases at play. Metacognitive training (MCT) [32] also is another evidence-based therapy that targets cognitive biases. Participants learn to modify biases that are linked to psychotic symptoms via trainings following specific modules [33]. Meta-analyses on MCT interventions have demonstrated small-to-moderate effects on positive symptoms [34, 35]. Cognitive bias modification training (CBMT) [36] also aims at modifying cognitive biases, although mostly attentional biases, specific to facial emotion recognition for instance. Other trainings exist, such as the Maudsley review training program (MRTP) [37], a computerized program that aims at decreasing Jumping to Conclusions via reasoning training (RT) [38], or Michael’s game, a card game designed to help people with psychotic disorders find alternative explanations for various situations that vary in paranoid intensity [39].

Interventions or trainings targeting cognitive biases may also exert positive effects on lack of clinical (unawareness of being ill) and cognitive (self-reflectiveness and self-certainty) insight [40, 41]. A recent meta-analysis also suggests that, overall, interventions targeting cognitive biases have a small, positive and statistically significant effect on the reduction of cognitive biases, a moderate significant positive effect on the improvement of psychotic symptoms, and a moderate significant positive effect on the improvement of patients’ insight levels [33].

Although cognitive biases have been linked to symptoms in most studies, few studies have looked at such biases transdiagnostically. Cognitive biases can be assessed through a variety of experimental tasks or self-reported questionnaires. The Beads task [42], for instance, has been extensively used for the Jumping to Conclusions bias [43–45].

Several questionnaires have been developed to assess various biases, including the Attributional Style Questionnaire [46], the Internal, Personal, and Situational Attributions Questionnaire [47], and the Ambiguous Intentions Hostility Questionnaire [48]. Most have a narrow focus and only target one type of cognitive bias, and as such do not provide a comprehensive assessment of an individual’s cognitive biases.

As reported by Peters and colleagues [49], several questionnaires evaluating the “Beck biases” (i.e., arbitrary interference, selective abstraction, magnification, minimisation, overgeneralisation, and personalisation) [50] are available in the mood disorder literature [51–57]. Peters and colleagues [49] thought that many of these questionnaires were less appropriate for people with a severe mental disorder or with occupational and social dysfunction, because they refer to work or social circumstances that might be quite different from theirs (e.g., “You noticed recently that a lot of your friends are taking up golf and tennis” [49, 56]. As a result, they developed the Cognitive Bias Questionnaire for psychosis (CBQp), a self-reported questionnaire that assesses cognitive biases (interpretation biases) and involves a wide range of thinking styles commonly observed among individuals with a psychotic disorder, as well as in individuals with other severe mental disorders. The CBQp is easy to use and was designed to be useful in both clinical and research settings. This comprehensive self-reported questionnaire enables the assessment of multiple biases concurrently, including: Catastrophising (predicting negative events in the future), Dichotomous Thinking (all-or nothing thinking), Emotional Reasoning (the use of subjective emotions to form conclusions); Intentionalising (thinking negative scenarios were committed on purpose), and Jumping to Conclusions (taking hasty decisions without having a sufficient amount of evidence).

Peters and colleagues [49]) validated their questionnaire in three populations, that is individuals with psychosis, with depression, and healthy controls. It has shown good internal consistency (α = 0.89) as a single factor and excellent test-retest reliability (α = 0.96). Scores on the CBQp questionnaire have been associated to those obtained on the Psychotic Symptoms Ratings Scales (PSYRATS) [58], the Beck Depression Inventory (BDI) [59], and the Beck Anxiety Inventory [60], providing good rationale for its concurrent validity.

Construct validity was investigated by correlating CBQp total and theme scores with the CST [51], and each of the five cognitive biases measured with its equivalent task or questionnaire (scores on the self items themes of the CST for Emotional Reasoning, the Beads Task [60] for Jumping to Conclusion, the Catastrophising Interview [61] for Catastrophising, the number of extreme responses on the Dysfunctional attitudes scale (DAS) [62] for Dichotomous Thinking, and the Ambiguous Intentions and Hostility Questionnaire (AIHQ) [48] for Intentionalising (accidental and ambiguous scenarios only). None of the CBQp individual bias scores were related to a task equivalent, apart from Emotional Reasoning and the CST self-based items. Moreover, the total CBQp score was correlated with the CST. The authors suggested that the demonstration of the construct validity of the individual CBQp biases was, to some degree, hampered by the lack of appropriate measures available in the literature [49].

Healthy controls scored significantly lower on the CBQp relative to the other group s[49].. Interestingly, CBQp total scores did not distinguish individuals with depressive and psychotic disorders, suggesting that these groups may present with similar cognitive biases. Based on their cluster factor analysis (CFA), the questionnaire seemed to assess a general thinking bias (1-factor), but the 2-factor model (assessing biases within two themes relating to psychosis; Anomalous Perception and Threatening Events) was the best fit when the factors were assumed to be related, and the 5-factor model factor (assessment of multiple biases) also showed a reasonable fit. The CBQp has been translated and validated in several languages, namely Flemish [63], Indonesian [64], Japanese [65], and Italian [66] (Pozza & Dettore, 2017). Thus far, the CBQp has yet to be validated in French.

Accordingly, the current study’s objective is to validate a French version of the CBQp and to replicate Peters and colleagues’ [42] findings by exploring transdiagnostic cognitive biases in individuals with psychotic disorders, individuals with mood disorder (depression), and in healthy controls. The study aims to: 1) translate the CBQp in French; 2) determine the validity and reliability of the French version; 3) verify its factorial structure; and 4) explore cognitive biases across diagnostic groups. Akin to Peters and colleagues (2014), we expected the French CBQp to be valid and reliable. Further, we expected that a one-factor or two-factor structure would be the best fit. We also expected that both the psychosis and depression groups would score higher on the CBQp relative to controls and that similar biases would be found across diagnostic groups.

## Methods

The study was cross-sectional, with participants answering the questionnaire only once.

### Participants

The Cognitive Bias Questionnaire for Psychosis (CBQp) [49] was administered to the control group (*N* = 663) either in paper format to several undergraduate classes at the University of Montreal or online through a survey website that was shared across several social media networks. Data from 84 participants were excluded due to too many missing answers. Therefore, 579 control participants were retained for statistical analysis. In addition to the controls, 62 participants with either a psychotic disorder (*N* = 30) or mood disorder (depression; *N* = 32) responded to the paper version. These participants were recruited through other ongoing studies in Quebec and France. Participants with a psychotic disorder were receiving services at a clinic for psychotic disorders and did not present with substance-induced psychosis. Participants with a mood disorder had been diagnosed with major depressive disorder and were currently on sick leave from work. Participants with a comorbid substance abuse disorder were not excluded, but those with a documented intellectual disability were. All participants provided socio-demographic as well as diagnostic information. The diagnoses were confirmed by a Structured Clinical Interview for DSM-5 (SCID) [67] or a psychiatric evaluation (*N* = 14) by a psychiatrist specializing in severe mental disorders.

### Measures

The CBQp is a 30-item questionnaire designed to assess cognitive biases. The questionnaire presents situations frequently encountered in everyday life, along with three response choices that reflect what an individual might think in these situations. Answers with an absence of bias merit one point, those with a presence of bias with some qualification receive two points, and those with a presence of bias are given a score of three points. The more biased the answers, the higher the score. The subscales are meant to measure: Intentionalising, Catastrophising, Jumping to Conclusion, Dichotomous Thinking and Emotional Reasoning biases. In addition, the questions were inspired by two themes relating to psychosis; Anomalous Perceptions (AP) and Threatening Event (TE).

The CBQp was translated into French from the original English version following a precise translation protocol based on international standards. Initially, we contacted the original authors of the CBQp and obtained their formal authorization to conduct the adaptation of the instrument into French. The CBQp was translated from English to French. The resulting French version was then translated back again to English by two experts. Those back-translations were compared to the original version by a third expert to identify and fix any discrepancies (reverse translation technique) [68]. The resulting French version was then checked by a translator to get the final version: “Le Questionnaire de Biais Cognitifs pour la psychose”. This translation technique was selected because it reduces the researcher’s bias, a translation that draws on the researcher’s culture and understanding. Translators tried to preserve semantic and inferential equivalence as much as possible.

### Statistics and procedures

Discriminant validity was determined via mean comparisons between groups for total scores and subscales scores measured. To test construct validity of the French version of the CBQp, confirmatory factor analyses were performed with R [69], according to the methods of Corbière & Larivière [70], who propose a probit model on categorical variables with a diagonally weighted least squares (DWLS) method. We investigated the one-, two-, and five-factor models to determine whether the model theorized by the original authors [49] would be preserved in our French version. A series of one-way ANOVAs were also conducted to compare the effect of diagnosis on the total score and each factorial solution of the CBQp. Since the assumption of homogeneity of variance was not met, ANOVAs were conducted with a Welch test, as well as Games-Howell post hoc procedure. Finally, cluster analyses were conducted to investigate cognitive biases across diagnostic groups (without the healthy controls). The variables used for the cluster analyses were the score for the five cognitive biases (Catastrophising, Dichotomous Thinking, Emotional Reasoning, Intentionalising and Jumping to Conclusions). Scores were standardized prior to the cluster analysis. We conducted a Clustering ensemble to ensure a more rigorous approach to cluster analyses [71, 72], which includes three methods (Hclust: Hierarchical clustering in R, with Ward Linkage [73]; the DB scan [74] and the K-means Clustering [75]). The data was then clustered using the number of times each pair of subjects had been classified together in the same group as a proximity value. The solution based on this last clustering was our final solution. We described each cluster profile and we compared them with chi-squared (for categorical variables) and ANOVAs (for continuous variables) based on diagnosis, age, gender, total cognitive bias score, and each of the cognitive bias scores. *Post-hoc* analyses with Bonferroni correction were conducted when a between-group difference was found.

Once the final cluster profile was selected, belonging to a particular cluster was used as the dependent variable in a multinomial logistic regression to test if diagnosis and demographics (age and gender) predicted cognitive bias profiles. A multinomial logistic regression model is appropriate instead of running three separate regression models because separate logistic regression models for each indicator variable are estimated simultaneously, allowing for mutually exclusive response categories to be analyzed without the need for overlapping reference categories [76, 77].

## Results

The socio-demographic and diagnostic information for each group (healthy control, psychosis group, and depression group) are reported in Table 1.

**Table 1:** Demographic and clinical information for the three groups

Characteristic	Control Group *N* = 576	Psychosis Group *N* = 31	Depression Group *N* = 31
Age, Mean (SD)	24.60 (7.0)	31.67 (6.3)	43.77 (10.6)
Gender, N (%)			
Women	215 (72.0%)	9 (29.0%)	27 (87.1%)
Men	158 (27.4%)	21 (67.7%)	4 (12.9%)
Other	2 (0.3%)	0 (0%)	0 (0%)
ND	1 (0.2%)	1 (3.2%)	0 (0%)
Principal Diagnosis, N (%)			
Major Depressive Disorder	–	–	31 (100.0%)
Bipolar Disorder	–	1 (3.2%)	–
Schizophrenia	–	25 (80.6%)	–
Schizoaffective Disorder	–	1 (3.2%)	–
Unspecified Psychotic Disorder	–	4 (12.9%)	–

### Ability to discriminate between diagnostic group and healthy controls

Total scores and subscale scores on the CBQp are shown next to those from Peters and colleagues [49] in Table 2. For all the subscales, our healthy control group consistently scored slightly higher (i.e., more biases) relative to that of Peters and colleagues [49], while our depression group consistently scored slightly lower. Our psychosis group showed comparable results to those in Peters and colleagues’ [49] study.

**Table 2:** Comparison between the French validation and the original study

	Control Group *N* = 576		Psychosis Group *N* = 31		Depression Group *N* = 31	
Scale	Current study	Original study	Current study	Original study	Current study	Original study
Total score	40.9 (4.6)	36.5 (2.7)	47.3 (8.9)	47.3 (10.4)	41.3 (7.0)	45.5 (9.4)
Theme						
Threatening Events	21.1 (3.0)	19 (1.7)	25.1 (5.5)	24.6 (6.0)	21.0 (3.7)	24.7 (5.9)
Anomalous Perceptions	19.8 (2.4)	17.5 (1.6)	22.2 (4.5)	22.7 (5.1)	20.4 (3.8)	20.8 (4.2)
Cognitive biase						
Intentionalising	7.7 (1.1)	7.3 (1.1)	8.7 (2.2)	8.8 (2.4)	7.5 (1.4)	7.7 (2.4)
Catastrophising	8.5 (1.5)	7.1 (0.9)	9.9 (2.6)	9.5 (2.4)	8.2 (1.6)	9.1 (2.1)
Dichotomous Thinking	7.8 (1.3)	6.5 (0.7)	9.0 (2.2)	8.8 (2.6)	7.9 (2.0)	9.5 (2.9)
Jumping to conclusions	9.4 (1.6)	8.5 (1.3)	10.6 (1.8)	10.7 (2.5)	9.3 (1.8)	10.9 (1.9)
Emotional Reasoning	7.6 (1.41)	7.2 (1.1)	9.1 (2.7)	9.4 (2.5)	8.4 (2.0)	8.3 (2.1)

Comparison between the scores of the current study on the French validation of the Cognitive Bias Questionnaire for psychosis (CBQ(p)) and the scores of the original study (Peters et al., 2014) across the three groups. Data presented as mean (standard deviation)

Overall, as can be observed in Table 3, the psychosis group scored higher than one or both comparison groups for each subscale and total score. No significant differences were found between psychosis and depression groups for subscales Anomalous Perception, Dichotomous Thinking and Emotional Reasoning, but the differences remained throughout between the psychosis and the control groups. No differences were noted between the depression and control groups.

**Table 3:** Effect of diagnosis on total score and each subscore of the CBQ(p)

Scale	Welch’ F	dfm, dfr	p	Difference (95%CI)
Total Score	7.92	2, 41.49	.001	
Psychosis vs. Control				6.45599* (2.4841-10.4279)
Depression vs. Control				.47045 (−2.6691-3.6100)
Psychosis vs. Depression				5.98554* (1.0780-10.8931)
Anomalous Perceptions	4.82	2, 41.48	.013	
Psychosis vs. Control				2.47581* (.4580-4.4936)
Depression vs. Control				.61636 (−1.0958-2.3286)
Psychosis vs. Depression				1.85945 (−.7036-4.4225)
Threatening events	8.09	2, 42.01	.001	
Psychosis vs. Control				3.99886* (1.5646-6.4332)
Depression vs. Control				−.14861 (−1.8277-1.5304)
Psychosis vs. Depression				4.14747* (1.2804-7.0145)
Catastrophising	4.98	2, 42.57	.011	
Psychosis vs. Control				1.42727* (.2807-2.5738)
Depression vs. Control				−.23079 (−.9737-.5121)
Psychosis vs. Depression				1.65806* (.3346-2.9815)
Dichotomous Thinking	5.15	2, 41.80	.010	
Psychosis vs. Control				1.25274* (.2920-2.2135)
Depression vs. Control				.16887 (−.7031-1.0409)
Psychosis vs. Depression				1.08387 (−.1700-2.3378)
Emotional Reasoning	7.65	2, 41.70	.001	
Psychosis vs. Control				1.52907* (.3485-2.7096)
Depression vs. Control				.85165 (−.0269-1.7302)
Psychosis vs. Depression				0.67742 (−.7491-2.1039)
Intentionalising	3.66	2, 41.68	.034	
Psychosis vs. Control				1.04078* (.0587-2.0228)
Depression vs. Control				−.19148 (−.8238-0.4409)
Psychosis vs. Depression				1.23226* (.965-2.3681)
Jumping to Conclusions	6.90	2, 43.24	.003	
Psychosis vs. Control				1.19300* (.4020-1.9840)
Depression vs. Control				−.13603 (−.9569-0.6849)
Psychosis vs. Depression				1.32903* (.2357-2.4224)

*p < .05

### Confirmatory factor analysis

#### Healthy control group

Confirmatory factor analysis revealed that the one-factor model explained 86.6% of the saturated model, with a Root Mean Square Error of Approximation (RMSEA) of .035, and a Percent Confidence Interval RMSE of (.030, .039). The two-factor model explained 88.2% of the saturated model, with a CFI of .882, a RMSEA of .033 and a Percent Confidence Interval RMSE of (.028, .037). The five-factor model explained 89.5% of the saturated model, with a RMSEA of .031 and a Percent Confidence Interval RMSE of (.026, .036; see Table 4).

**Table 4:** Goodness of Fit for the CFA, comparison between the French version and the original study

	Current study (control group)					Current study (psychosis and depression groups)					Original study (psychosis group)				
Model	CFI	RMSEA	χ2	*p*	IBF	CFI	RMSEA	χ2	*p*	IBF	CFI	RMSEA	χ2	*p*	IBF
five-factor model; Independent factors	.179	.086 (.082-.082)	2126.257	< .001	–	.207	.256 (.244-.268)	1808.187	< .001	–	.464	.083 (.077-.088)	1133.99	< .001	–
five-factor model; Related factors	.895	.031 (.026-.036)	615.071	< .001	.656-1.359	.922	.081 (.063-.098)	533.724	< .001	.709-1.062	.933	.030 (.019-.038)	485.90	< .001	.89-.98
two-factor model; Independent factors	.598	.060 (.056-.064)	1246.704	< .001	–	.610	.179 (.167-.192)	1094.894	< .001	–	.779	.061 (.054-.067)	677.21	< .001	–
two-factor model; Related factors	.882	.033 (.028-.037)	650.975	< .001	.745	.926	.078 (.059-.095)	535.010	< .001	.832	.969	.022 (.001-.024)	92.44	.201	.77
one-factor model	.866	.035 .030-.039)	685.464	< .001	–	.920	.081 (.063-.098)	547.315	< .001	–	.934	.029 (.019-.037)	494.09	.002	–

*CFA* Confirmatory factorial analysis (CFA), comparison between the French version and the original study (Peters et al., 2014)

*CFI* Comparative Fit Index

RMSEA, Root Mean Square Error of Approximation, *IBF* Intercorrelations Between Factors

#### Clinical groups

Regarding the factorial validation with the clinical groups (pooled together), the one-factor model explained 92.0% of the saturated model, with a RMSEA of .081 and a Percent Confidence Interval RMSE of (.063, .098). The two-factor model explained 92.6% of the saturated model, with a RMSEA of .078 and a Percent Confidence Interval RMSE of (.059, .095). The five-factor model explained 92.2%of the saturated model, with a RMSEA of .081 and a Percent Confidence Interval RMSE of (.063, .098; See Table 4).

### Cognitive biases profiles

**Fig. 1:**
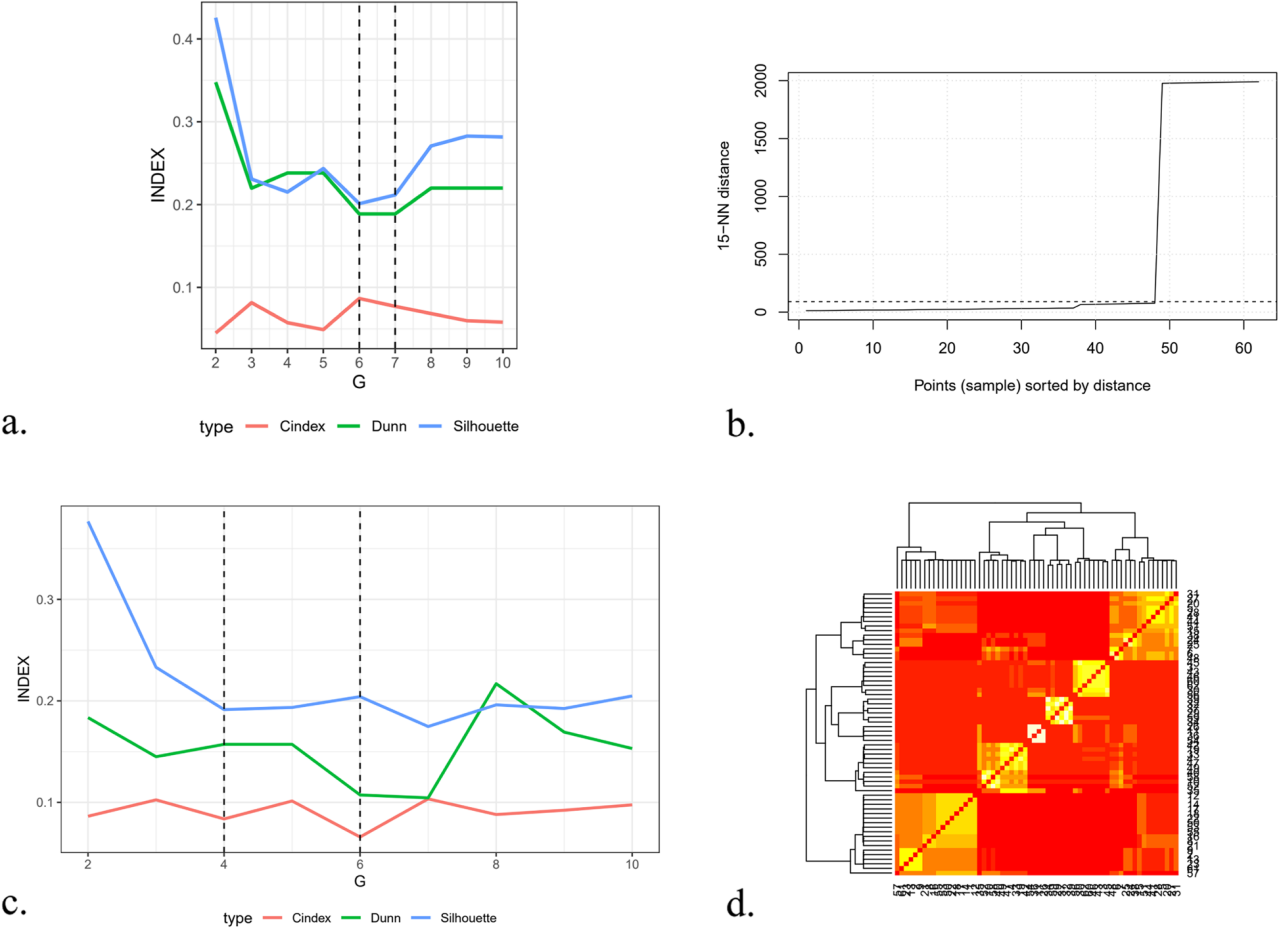
Cluster analyses **a.** Hierarchical clustering in R, with Ward Linkage **b.** DB scan **c.** K-means Clustering **d.** Clustering ensemble

**Fig. 2:**
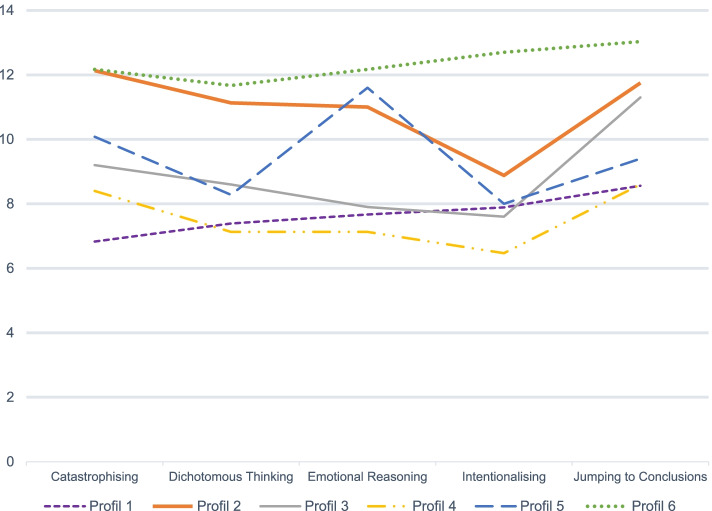
Cognitive biases Profiles

The first cluster analysis, the Hierarchical clustering, suggested a six- to seven-cluster solution (see Fig. 1a). The second cluster analysis, the DB-scan, favored a solution with only one group (see Fig. 1b), thus it was not useful for our objective. The third cluster analysis, the K-means clustering, suggested four to six profiles (see Fig. 1c.). The six-profile solution emerged from the final cluster analysis, the Clustering ensemble (see Fig. 1d). For a visual presentation of the cognitive biases’ profiles, see Fig. 2. For descriptive information for each profile, see Table 5.ANOVAs showed that the association between total score and profiles was significant, F(5,56) = 118.28, *p* < .001, as for the association between each cognitive biases scores and profiles (Intentionalising: F(5,56) = 23.83, *p* < .001; Catastrophising: F(5,56) = 36.13, *p* < .001); Dichotomous Thinking: F(5,56) = 17.63, *p* < .001); Emotional Reasoning: F(5,56) = 21.36, *p* < .001); Jumping to Conclusions: F(5,56) = 33.67, *p* < .001). See S.2 to S.8 in Supplementary Materials for the post hoc analysis of each ANOVA. Bonferroni correction was applied to post hoc analyses (*p* < .003). Cognitive bias scores for each profile will be presented by profiles, with the profiles with highest total score presented first. Scores results are reported as (Mean ± SD).

**Table 5:** Demographics, diagnosis, and CBQ(p) scores for the six profiles

Variable	Profile 1	Profile 2	Profile 3	Profile 4	Profile 5	Profile 6
Participants, N (% across profiles)	18 (29%)	8 (12.9%)	10 (16.1%)	15 (24.2%)	5 (8.1%)	6 (9.7%)
Age, Mean (SD)	45.61 (9.35)	30.57 (5.62)	34.80 (7.64)	38.40 (12.44)	33.00 (8.00)	30.50 (4.72)
Gender, N (% within-group) ±						
Women	10 (55.6%)	5 (71.4%)	5 (50%)	12 (80%)	3 (60%)	1 (16.7%)
Adjusted Residual	−.4	.7	−.6	1.9	.0	−2.2
Men	8 (44.4%)	2 (28.6%)	5 (50%)	3 (20%)	2 (40%)	5 (83.3%)
Adjusted Residual	.4	−.7	.6	−1.9	.0	2.2
Diagnosis category (% within group)						
Depression	13 (72.2%)	2 (25%)	3 (30%)	10 (66.7%)	2 (40%)	1 (16.7%)
Adjusted Residual	2.2	−1.5	−1.4	1.5	−0.5	−1.7
Psychosis	5 (27.8%)	8 (75%)	7 (70%)	5 (33.3%)	3 (60%)	5 (83.3%)
Adjusted Residual	−2.2*	1.5	1.4	−1.5	0.5	1.7
Cognitive Bias Score, Mean (SD)						
Total Score	38.33 (1.91)	54.88 (1.55)	44.63 (2.57)	37.75 (2.87)	47.43 (1.22)	61.68 (4.97)
Intentionalising	7.89 (1.08)	8.88 (.64)	7.60 (1.07)	6.47 (.74)	8.00 (1.00)	12.70 (1.38)
Catastrophising	6.83 (.71)	12.13 (.83)	9.20 (1.55)	8.40 (1.40)	10.08 (1.06)	12.17 (2.04)
Dichotomous Thinking	7.39 (.98)	11.13 (1.13)	8.60 (1.51)	7.13 (.74)	8.28 (.83)	11.67 (3.14)
Jumping to Conclusion	8.56 (.78)	11.75 (1.67)	11.30 (.95)	8.60 (.83)	9.40 (1.14)	13.03 (.64)
Emotional Reasoning	7.67 (.84)	11.00 (2.33)	7.90 (1.29)	7.13 (1.25)	11.60 (.89)	12.17 (2.23)

*p < .05

±one participant did not answer this question and was removed from the gender analysis

Profile 6 had higher total score (61.38 ± 4.97) and individual cognitive bias score than every other profile. Most differences were significant at *p* < .05 and survived the Bonferroni correction.

Profile 2 had a higher total score (54.88 ± 1.55) than Profiles 1, 3, 4, and 5 (*ps* < .003). Profile 2 had higher results for every cognitive biases, with Intentionalising being his lowest score (8.88 ± 0.64). Intentionalising was still significantly higher for Profile 2 than for Profiles 1, 3 (*ps* < .05) and 4 (*p* < .003).

Profile 5 had a total score of 47.43 ± 1.22, significantly higher than Profiles 1 and 4, but significantly lower than Profiles 2 and 6 (*ps* < .003). The total score of Profile 5 was not significantly different from that of Profile 3 (*p* > .05). The highest score was for Emotional Reasoning (11.30 ± 0.89).

Profile 3 had a total score of 44.63 ± 2.57, lower than Profiles 2 and 6 (*p* < .003), but higher than Profile 4 (*p* < .003), and similar to Profile 5 (*p* > .05). The highest score for Profile 3 is Jumping to Conclusion.

Profile 1 had lower total score (38.33 ± 1.91) than every other profile (*p* < .003) except for Profile 4 (p > .05). The highest score of Profile 1 is Jumping to Conclusion, but visually looking at the data, it is still lower than for every other profile (significantly lower than Profiles 2, 3 and 6, *ps* < .003).

Profile 4 had a total score of 37.75 ± 2.87, similar to profile 1 (*p* > .05), and significantly lower than every other profile (*p* < .003). Visually, the highest scores seem to be for Catastrophizing and Jumping to Conclusion.

An ANOVA showed a significant association between age and profile, *F*(5.55) = 4.63, *p* = .001 (see Table 5). Post hoc analysis indicated a difference between Profile 1 and every other profile. However, only the difference between Profile 1 and both the Profiles 2 and 6 survived the Bonferroni correction, with Profile 1 being older (45.61 ± 9.35 years old) relative to Profile 2 (30.57 ± 5.62; *p* = .009 years) and Profile 6 (30.50 ± 4.72; *p* = .016). No significant differences were observed between other profiles based on this variable, *ps* > .05 (see S.1 in Supplementary Materials).

Chi-squared tests showed that the association between gender and profile was not significant, X^2^ (61) = 8.05, *p* = .153. However, visually, we can observe a greater proportion of women in Profile 4 (80.0%, adjusted residual = 1.9) and a greater proportion of men in Profile 6 (83.3%, adjusted residual = 2.2; See Table 5). However, the association between diagnosis and profiles was significant, *X*^*2*^ (62) = 11.69, *p* = .039. Profile 1 had a significantly greater proportion of individuals with depression than other profiles (72.2%, adjusted residuals = 2.2). Only the difference with Profile 2 survived to post hoc analysis (*p* < .05), but did not survive the Bonferroni correction (see S.2 in Supplementary Material). Although not significant, we can visually observe that Profile 4 also had a greater proportion of individuals with depression (66.7%, adjusted residuals = 1.5), and that Profiles 2, 3, and 6 have a greater proportion of individuals with psychosis (respectively, 75%, adjusted residuals = 1.5; 70%, adjusted residual = 1.4; and 83.3%, adjusted residuals = 1.7). We can see that Profile 4 had a similar proportion of men and women (see Table 5).

Since the psychosis group and the depression group were not balanced in terms of age and gender, we performed stratifications before running the multinomial regression. However, there were too few individuals in each cell for interpretation, and the results were not significant, *p* > .05. Therefore, we ran the multinomial regression without stratifications. Among demographic variables (diagnosis, age, and gender), only age continued to be statistically different among the Profiles when variables are considered together, *p* < .01.

## Discussion

The current study aimed at replicating Peters and colleagues’ [49] study with a French sample. Compared to Peters and colleagues’ results [49], our control group showed higher scores, our depression group showed lower scores, and our psychosis group showed similar scores. It is not surprising that our control group had higher scores since most participants in this group were recruited from university courses. That is, several studies indicate that university students have a higher prevalence of depressive symptoms than the general population. A survey conducted by the National College Health Assessment with 43,780 students indicated that 44% of respondents have experienced symptoms of depression in the last 12 months [78]. According to a survey of 10,000 students from the student association *Fédération des Associations Étudiantes du Campus de l’Université de Montréal (FAÉCUM)*, 22% reported moderate-to-severe depressive symptoms or severe depression, 5.8% reported symptoms of exhaustion, and 7.8% reported having seriously thought about killing themselves in the past 12 months [79]. Furthermore, our depression group had lower scores relative to those from Peters and colleagues’ [49] study. This may be explained by the fact that individuals in our depression group were recruited through another study where participants had been on sick leave due to depression and were now returning to work. Although some participants still presented with severe depressive symptoms, most had experienced a reduction in symptoms. Furthermore, our results reflect the transdiagnostic character of cognitive biases; an individual’s score does not only depend on their diagnosis, but also on their current condition and level of distress. In their meta-analysis about attributional biases and the capacity to discriminate internal and external events, Brookwell, Bentall & Varese [80] suggest that the cognitive biases they studied were not diagnostic-specific, but could be accounted for by the presence of hallucinatory experiences. Future studies should study cognitive bias profiles in relation to specific symptoms, including symptom severity, rather than diagnoses.

Based on prior studies [49, 63, 65], we expected that the one-factor structure would prevail over the two- or five-factor structure, for both the control and clinical groups. This hypothesis was confirmed for the control group, suggesting that biases tend to co-exist. For example, an individual with a strong Catastrophising bias is likely to also have a strong Intentionalising bias. This does not mean that the questionnaire measures a single concept, but rather, that they are tightly connected. For the clinical group, all three models were acceptable, although the small sample resulted in less-than-ideal RMSEA.

The three-step cluster analyses aimed to determine profiles of individuals based on their cognitive biases, assessed by the CBQp and to compare the resulting clusters on sociodemographic (i.e., age, gender) and clinical (i.e., diagnosis) characteristics. Consistent with our hypothesis, more than two clusters emerged, suggesting that individuals with similar diagnoses do not score the same on all cognitive biases, and that individuals with different diagnoses might have similar cognitive biases. The clusters indicated that although some people seem to score similarly across biases, suggesting a tendency to have many biases together (as the one-factor model suggests), others have a strong tendency for presenting with some biases above all others.

All profiles were significantly different in terms of cognitive biases, yet not surprising since clustering was performed on those specific variables. It is interesting to notice that individuals with some of the highest bias scores are younger (Profiles 2 and 6), whereas those with some of the fewest biases (Profile 1) are older. It is possible that these biases tend to decrease as individuals age; however, it is also plausible that this finding could be better explained by the fact that Profile 1 had a greater percentage of individuals with depression, and that our depression group was older than our psychosis group. Because age was the only variable that still predicted profiles after the multinomial regression, a larger sample will be needed to truly determine whether diagnosis or gender are significant predictors of clusters.

Our results suggest that, although some biases might be more present in individuals with specific symptoms (e.g., Jumping to Conclusions is more typical in profiles with more individuals with psychosis) or from a specific gender (e.g., Emotional Reasoning is more prevalent in profiles with more women), these are not specific to these individuals and can be found in other profiles as well.

### Limitations

Several limitations of the present study must be acknowledged. First, the healthy control group was homogeneous and included only university students – different results might be obtained with a different sample. Another limitation is that some diagnoses were not confirmed by a structured clinical interview. For the validation of the factorial structure of the questionnaire for the clinical group, a larger sample would be needed to confirm our results. Furthermore, for the cluster analysis, there were significant demographic differences between groups, with individuals in the depression group being older than those of the psychosis group. Individuals in the depression group were mostly female whereas individuals in the psychosis group were mostly males. Although this represents a clinical reality, it may have biased our results. Because our sample was too small to use stratifications, we can only conclude about the effect of age on the profiles.

### Research and clinical implication

This study demonstrated the validity of the French version of the CBQp, therefore allowing clinicians and researchers to easily assess cognitive biases in French-speaking participants. It is a simple and efficient questionnaire that can be used evaluate whether participants receiving CBTp, for instance, have experienced changes in their cognitive biases following the therapy. Our findings highlight the interdependence of different cognitive biases and could be indicative of a common cognitive process leading to the development of several biases. Moritz and al [81]. also suggest that cognitive mechanisms for delusions only partially overlap and recommend continuing studying and targeting specific biases via metacognitive training.

According to Garety and Freeman [82], cognitive biases contribute to maintaining psychotic symptoms such as delusions. For instance, a dichotomous style of thinking would seem to favor the maintenance of delusions, while Jumping to Conclusions would favor both the emergence and the maintenance of psychotic symptoms [83]. To date, most studies have focused on a specific cognitive bias, whereas our study suggests that studying or addressing multiple biases together might be useful. From our clinical experience, individuals in group CBT for psychosis for instance, benefit from learning about and recognizing their own cognitive biases, as this helps them take a step back from distressing thoughts and work on strategies to verify and modify their current beliefs.

## Conclusion

The current findings support the validity of the French translation of the CBQp. We obtained results similar to the original validation study of the questionnaire. The authors of the original CBQp have recommended using one-, two-, or five-factor structure. While the results of our study are similar, the 5-factor solution is clinically more interesting. Our cluster analyses overall support the transdiagnostic presence of cognitive biases.

## Supplementary Information

**Additional file 1.**

## Data Availability

The authors do not have ethical approval to share the raw data that support the findings. However, the datasets used and/or analyzed during the current study available from the corresponding author on reasonable request.
