# Supplementary material for: Further validation of the Cognitive Biases Questionnaire for psychosis
Source: BMC Psychiatry. 2022 Aug 19;22:560. doi: 10.1186/s12888-022-04203-8 (PMC9392283; doi:10.1186/s12888-022-04203-8)
Supplement: Supplementary file 1 — Additional file 1. [file 12888_2022_4203_MOESM1_ESM.docx]

**Supplementary Materials**

S.1 One-way ANOVA Post Hoc tests for age, mean difference between profiles

S.2 Fisher’s Exact test *p*-values for diagnosis (two-sided)

S.3 One-way ANOVA *post hoc* tests for Total score, mean difference between profiles
S.4 One-way ANOVA *post hoc* test for Intentionalising, mean difference between profiles
S.5 One-way ANOVA *post hoc* test for Catastrophising, mean difference between profiles
S.6 One-way ANOVA *post hoc* test for Dichotomous Thinking, mean difference between profiles
S.7 One-way ANOVA *post hoc* test for Emotional Reasoning, mean difference between profiles
S.8 One-way ANOVA *post hoc* test for Jumping to Conclusion, mean difference between profiles

**S1.** One-way ANOVA P*post hoc* tests for age, mean difference between profiles in years

| **Profiles** | **1** | **2** | **3** | **4** | **5** | **6** |
| --- | --- | --- | --- | --- | --- | --- |
| **1** |  | 15.040^**^ | 10.811* | 7.211* | 12.611* | 15.111^**^ |
| **2** |  |  | -4.229 | -7.829 | -2.429 | 0.071 |
| **3** |  |  |  | -3.600 | 1.800 | 4.300 |
| **4** |  |  |  |  | 5.400 | 7.900 |
| **5** |  |  |  |  |  | 2.500 |
| **6** |  |  |  |  |  |  |

**p* < .05

** significant after Bonferroni correction, *p* < .003

**S.2.** Fisher’s Exact test *p*-values to compare the profiles on diagnosis (two-sided)

| **Profiles** | **1** | **2** | **3** | **4** | **5** | **6** |
| --- | --- | --- | --- | --- | --- | --- |
| **1** |  | .038* | .050 | 1.000 | .297 | .050 |
| **2** |  |  | 1.000 | .089 | 1.000 | 1.000 |
| **3** |  |  |  | .111 | 1.000 | 1.000 |
| **4** |  |  |  |  | .347 | .063 |
| **5** |  |  |  |  |  | .545 |
| **6** |  |  |  |  |  |  |

**p* < .05

** significant after Bonferroni correction, *p* < .003

**S.3.** One-way ANOVA *post hoc* tests for Total score, mean difference in scores between profiles

| **Profiles** | **1** | **2** | **3** | **4** | **5** | **6** |
| --- | --- | --- | --- | --- | --- | --- |
| **1** |  | -16.54** | -6.30** | .58 | -9.09** | -23.34** |
| **2** |  |  | 10.24** | 17.12** | 7.45** | -6.83** |
| **3** |  |  |  | 6.88** | -2.79 | -17.04** |
| **4** |  |  |  |  | -9.67** | -23.92** |
| **5** |  |  |  |  |  | -14.25** |
| **6** |  |  |  |  |  |  |

**p* < .05

** significant after Bonferroni correction, *p* < .003

**S.4.** One-way ANOVA *post hoc* test for Intentionalising, mean difference in scores between profiles

| **Profiles** | **1** | **2** | **3** | **4** | **5** | **6** |
| --- | --- | --- | --- | --- | --- | --- |
| **1** |  | -.99* | .29 | 1.42** | -.11 | -4.81** |
| **2** |  |  | 1.28* | 2.41** | .88 | -3.83** |
| **3** |  |  |  | 1.13* | -.40 | -5.10** |
| **4** |  |  |  |  | -1.53* | -6.23** |
| **5** |  |  |  |  |  | -4.70** |
| **6** |  |  |  |  |  |  |

**p* < .05

** significant after Bonferroni correction, *p* < .003

**S.5.** One-way ANOVA *post hoc* test for Catastrophising, mean difference in scores between profiles

| **Profiles** | **1** | **2** | **3** | **4** | **5** | **6** |
| --- | --- | --- | --- | --- | --- | --- |
| **1** |  | -5.29** | -2.37** | -1.57** | -3.25** | -5.33** |
| **2** |  |  | 2.93** | 3.73** | 2.05* | -.04 |
| **3** |  |  |  | .80 | -.88 | -2.97** |
| **4** |  |  |  |  | -1.68* | -3.77** |
| **5** |  |  |  |  |  | -2.09* |
| **6** |  |  |  |  |  |  |

**p* < .05

** significant after Bonferroni correction, *p* < .003

**S.6.** One-way ANOVA *post hoc* test for Dichotomous Thinking, mean difference in scores between profiles

| **Profiles** | **1** | **2** | **3** | **4** | **5** | **6** |
| --- | --- | --- | --- | --- | --- | --- |
| **1** |  | -3.74** | -1.21* | .26 | -.89 | -4.28** |
| **2** |  |  | 2.53** | 3.99** | 2.85** | -.54 |
| **3** |  |  |  | 1.47* | .32 | -3.07** |
| **4** |  |  |  |  | -1.15 | -4.53** |
| **5** |  |  |  |  |  | -3.39** |
| **6** |  |  |  |  |  |  |

**p* < .05

** significant after Bonferroni correction, *p* < .003

**S.7.** One-way ANOVA *post hoc* test for Emotional Reasoning, mean difference in scores between profiles

| **Profiles** | **1** | **2** | **3** | **4** | **5** | **6** |
| --- | --- | --- | --- | --- | --- | --- |
| **1** |  | -3.33** | -.23 | .53 | -3.93** | -4.50** |
| **2** |  |  | 3.10** | 3.87 | -.60 | -1.17 |
| **3** |  |  |  | .77 | -3.70** | -4.27** |
| **4** |  |  |  |  | -4.47* | -5.03* |
| **5** |  |  |  |  |  | -.57 |
| **6** |  |  |  |  |  |  |

**p* < .05

** significant after Bonferroni correction, *p* < .003

**S.8.** One-way ANOVA *post hoc* test for Jumping to Conclusion, mean difference in scores between profiles

| **Profiles** | **1** | **2** | **3** | **4** | **5** | **6** |
| --- | --- | --- | --- | --- | --- | --- |
| **1** |  | -3.19* | -2.74* | -.04 | -.84 | -4.48* |
| **2** |  |  | .45 | 3.15** | 2.35** | -1.28* |
| **3** |  |  |  | 2.70** | 1.90** | -1.73** |
| **4** |  |  |  |  | -.80 | -4.43** |
| **5** |  |  |  |  |  | -3.63** |
| **6** |  |  |  |  |  |  |

**p* < .05

** significant after Bonferroni correction, *p* < .003
